# Anatomically Guided Deep Learning System for Right Internal Jugular Line (RIJL) Segmentation and Tip Localization in Chest X-Ray

**DOI:** 10.3390/life15020201

**Published:** 2025-01-29

**Authors:** Siyuan Wei, Liza Shrestha, Gabriel Melendez-Corres, Matthew S. Brown

**Affiliations:** Center for Computer Vision and Imaging Biomarkers, Department of Radiological Sciences, David Geffen School of Medicine at UCLA, University of California, Los Angeles, CA 90095, USA; weis97@g.ucla.edu (S.W.); lshrestha@mednet.ucla.edu (L.S.); gmelendezcorres@mednet.ucla.edu (G.M.-C.)

**Keywords:** artificial intelligence (AI), deep learning, segmentation, chest X-ray, catheters, anatomical landmark, cognitive AI

## Abstract

The right internal jugular line (RIJL) is a type of central venous catheter (CVC) inserted into the right internal jugular vein to deliver medications and monitor vital functions in ICU patients. The placement of RIJL is routinely checked by a clinician in a chest X-ray (CXR) image to ensure its proper function and patient safety. To reduce the workload of clinicians, deep learning-based automated detection algorithms have been developed to detect CVCs in CXRs. Although RIJL is the most widely used type of CVCs, there is a paucity of investigations focused on its accurate segmentation and tip localization. In this study, we propose a deep learning system that integrates an anatomical landmark segmentation, an RIJL segmentation network, and a postprocessing function to segment the RIJL course and detect the tip with accuracy and precision. We utilized the nnU-Net framework to configure the segmentation network. The entire system was implemented on the SimpleMind Cognitive AI platform, enabling the integration of anatomical knowledge and spatial reasoning to model relationships between objects within the image. Specifically, the trachea was used as an anatomical landmark to extract a subregion in a CXR image that is most relevant to the RIJL. The subregions were used to generate cropped images, which were used to train the segmentation network. The segmentation results were recovered to original dimensions, and the most inferior point’s coordinates in each image were defined as the tip. With guidance from the anatomical landmark and customized postprocessing, the proposed method achieved improved segmentation and tip localization compared to the baseline segmentation network: the mean average symmetric surface distance (ASSD) was decreased from 2.72 to 1.41 mm, and the mean tip distance was reduced from 11.27 to 8.29 mm.

## 1. Introduction

Central venous catheters (CVCs) are commonly used for venous access in clinical settings, particularly for patients requiring intensive care. These catheters are placed into central or peripheral veins and advanced to the superior vena cava (SVC) or right atrium to deliver medications and monitor the hemodynamic status. Based on access sites and intended duration, CVCs are categorized into several types [1]: for temporary venous access, the preferred insertion points are the internal jugular, subclavian, and common femoral veins; for mid- to long-term use, peripherally inserted central catheters (PICCs) are applied through the basilic and brachial veins.

A right internal jugular line (RIJL) is a CVC that is inserted into the right internal jugular vein (IJV). Since the right IJV has a much larger diameter than the left and provides a straighter path to the SVC, a right-sided approach minimizes the risk of severe complications such as pneumothorax and arterial bleeding [2]. As a result, RIJLs are the most common type of CVCs [3].

The correct positioning of CVCs is crucial, as improper placement can lead to serious, potentially life-threatening complications. To ensure patient safety and optimize treatment outcomes, a chest X-ray (CXR) is typically acquired after each CVC insertion. A prompt and accurate interpretation of the CXR by a radiologist is essential to confirm the correct catheter placement. While verifying CVC placement and repositioning, if necessary, are standard practices, reviewing CXRs on a large scale can be labor-intensive and may delay critical care. Computer-aided detection (CAD) systems offer a solution by streamlining the interpretation process and minimizing turnaround times.

**Figure 1:**
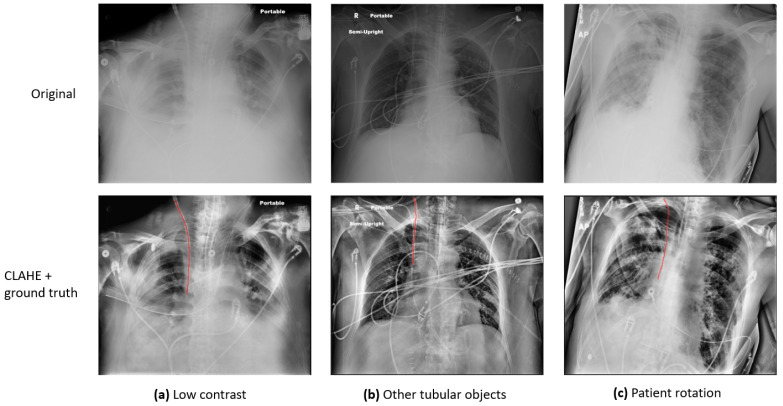
Sample CXR images to demonstrate the variation of image quality. The first row presents the original images. The second row presents the processed ones with CLAHE overlaid with the ground truth label, marked with red lines.

Previous studies [4,5] have identified several major challenges in the automatic detection and evaluation of CVCs in CXR images: (1) Some CXRs are of low pixel contrast. (2) The CVC is a narrow, tubular structure that occupies a disproportionately small area of the full CXR image, which makes it challenging to detect. (3) In addition to the CVC, other tubes and anatomical structures with similar appearances may also be present in a single CXR, and they can be difficult to distinguish. (4) Since the CXRs are acquired on ICU patients, the images might be rotated, adding another degree of variation to the image quality. Figure 1 presents a few sample images demonstrating these variations: (a) presents an image of low contrast and preprocessing is required to enhance the visibility of anatomy and foreign objects; (b) exhibits an image in which there are multiple tubular objects, which can be indistinguishable from one another; (c) shows a rotated patient, which can significantly affect clinical decisions. To address the challenges, various investigations have been conducted, and a detailed review is provided in Section 2: Literature Review.

As the most common CVC type, RIJLs are prevalent in both clinical practice and public CXR catheter datasets. All the previous studies, mentioned in Section 2, contributed to different aspects of the detection and evaluation of CVCs in CXRs. However, there is a paucity of investigations regarding the accurate segmentation of RIJLs, which is a prerequisite for placement verification and subsequent translation into the clinic. In the field of deep learning, various techniques have been developed for image segmentation: fully convolutional networks (FCNs), U-Net and its variants, transformer-based models such as vision transformers, etc.

Although deep learning has demonstrated exceptional performance, its black-box nature and lack of explainability continue to raise concerns regarding its reliability. Incorporating human knowledge and reasoning is particularly essential in the field of medical image analysis to enhance trust and interpretability. In this study, we propose an anatomical landmark-guided system to segment the course of the RIJL and localize the tip in CXRs, implemented on an open-source Cognitive AI software platform. In particular, the proposed system includes three components: (1) A convolutional neural network (CNN) configured by nnU-Net [6], a medical image segmentation framework that has achieved state-of-the-art performance and robustness across various imaging modalities. (2) Trachea-guided subregion extraction to define the most relevant area for the RIJL, by which we demonstrate the effectiveness of embedding human knowledge (e.g., “an RIJL should be placed 3–7 cm to the right of the trachea”; “an RIJL is 5 to 10 cm long”) into a deep learning algorithm. (3) A postprocessing algorithm that actively adapts to the shape of the RIJL and connects all the line fragments to enhance the final segmentation. The goal of this work is to improve the accuracy of the segmentation and tip localization of RIJLs in CXRs. Our experimental results have proved the effectiveness of the proposed methodology. To summarize, this study’s contributions are as follows:

(1) To our knowledge, this is the first study focused on segmenting the course of RIJL and localizing the tip in CXRs. As the most common type of CVCs, RIJL has a crucial role in the clinic and needs substantial effort to detect with accuracy and reliability.
(2) We have used CNN configurations from nnU-Net, for the first time, to segment the RIJL. Unlike many other deep learning segmentation models that require time-consuming, labor-intensive manual parameter tuning, nnU-Net provides a systematic, automated approach to configure the entire segmentation network and has proven its superior performance.
(3) We incorporated anatomical landmark segmentation to provide contextual information for segmenting relevant objects and presented improved results compared to a baseline segmentation. We have demonstrated that, for complicated tasks such as RIJL segmentation, it is beneficial to embed human knowledge and reasoning into the development strategies of algorithms.

The remainder of this article is organized as the following: Section 2 summarizes previous studies related to catheter segmentations in CXRs; Section 3 provides a detailed description of the methodology; Section 4 presents our experimental results; Section 5 discusses some interesting points and limitations in this study and offers future directions; Section 6 concludes the article.

## 2. Literature Review

With the rapid advancement of artificial intelligence in recent years, some studies have developed deep learning algorithms to detect and evaluate the placement of CVCs in CXR images. Ref. [4] developed a segmentation with U-Net [7] followed by classification with the EfficientNet [8] approach to check the positioning status of catheters in CXRs. Ref. [9] used transfer learning with ResNet to detect abnormal catheter positioning. To categorize CVC subtypes, a study [10] has combined deep neural network segmentation outputs with conventional machine learning on features extracted from spatial shape descriptors, which improves detection and classification. Specifically, the CVC and relevant anatomies were segmented with trained U-Net CNNs. The features were extracted from the segmentation outputs of the CVC and anatomies and then input to a random forest classifier to identify the CVC type. Henderson et al. [11] used a multi-label approach to classify different types of catheters and achieved good performance. A commercially available deep learning application for detecting and classifying the positions of various catheters in CXRs was evaluated in [12]. This study also highlighted the issue of hidden stratification, a significant challenge that researchers and developers must address when translating algorithms into clinical practice. Given the scarcity of data in clinical medicine, data collection remains a challenge for many researchers. Yi et al. [13] developed a catheter detection deep learning algorithm in pediatric X-ray with data synthesization to enhance the training dataset. As mentioned before, the presence of other tubular devices in a single CXR can be misleading, and it is essential to distinguish them. An instance segmentation approach was implemented in [14] to segment the courses of multiple catheters and resolve the intersection area of superimposed catheters. This study employed an HR-Net-based [15] multi-task network that has two branches: a segmentation branch to delineate the pixels of a catheter and an embedding branch which was trained to map pixels to a 3D feature space, known as pixel embeddings, where pixels belonging to different instances are far apart. The two branches were combined to assign pixels to individual catheters and resolve intersections.

To improve the accuracy and reliability of CVC detection, additional information from both anatomical structures and catheters in the CXR needs to be integrated into the prediction model. Therefore, several studies [16,17,18] have developed models to segment relevant anatomical regions for optimal CVC tip placement, trace the CVC course, and localize the tip position to evaluate CVC placement. Specifically, these models first segment the CVC course and the relevant anatomical regions. The tip position is then extracted from the course, and a tip-to-landmark metric is calculated to assess its placement, using soft intersection [16] and absolute distance [17,18], respectively. Both [16,17] used a U-Net to segment the CVC and relevant anatomical regions and an EfficientNet to classify the placement. In [18], U-Net CNNs were used to segment relevant anatomical regions in CXR and estimate the tip of CVC and tracheal tubes, respectively, followed by the positioning assessment of the tubes.

Since the position of the CVC tip depends on its course, accurate segmentation of the course becomes essential. Thus, segmentation networks have therefore been utilized to delineate the course and extract the tip of peripherally inserted central catheters (PICCs) in CXRs. In [19], a region of interest (ROI) relevant to the PICC tip was segmented to enhance the tip localization derived from the PICC segmentation; FCNs [20] were utilized to segment the PICC and the PICC ROI region. To address the issue of line fragments in the CNN output, a multi-stage model was proposed in [21], consisting of a patch-based segmentation network and a line fragment reconnection network, incorporating fully convolutional DenseNet, to improve PICC segmentation performance. Due to the small footprint of the CVC in a CXR, foreground pixels are likely to be ignored during the CNN training process, leading to a class imbalance problem. To address this, in [5], a class frequency weighted loss function was implemented to train a U-Net to segment RIJL in CXRs. Notably, instead of the commonly used dense segmentation approach, a point-based coordinate encoding scheme was proposed in [22] to improve the connectivity when tracking the course of CVCs and other tubes in CXRs.

Since course-dependent tip localization can be unreliable with inaccurate course segmentation, some studies have approached tip localization as an independent object detection problem. A method was developed in [23], combining key point detection and probabilistic constellation modeling, to identify the CVC tip and anatomical landmarks, enabling the detection of CVC tip malposition. In [24], a multi-tasking system was designed, incorporating a U-Net to segment the PICC and a Faster R-CNN [25] to detect the tip. In this study, a small test set (*N* = 20) was used to evaluate tip detection performance by calculating the intersection over union (IoU) between the bounding boxes of the ground truth and predictions. A more extensive study was conducted in [26] in which a CVC tip detection framework was proposed, comprising a modified High-Resolution Net [27], a segmentation supervision module, and a deconvolution module. The tip detection model was trained and tested on a public dataset [28], with the Euclidean Distance in pixel [29] and the percentage of correct key points [30] as evaluation metrics.

## 3. Materials and Methods

In this section, the proposed methodology for RIJL segmentation and tip localization in CXRs is explained. The segmentation system consists of a deep convolutional neural network (CNN) configured by nnU-Net, a spatial reasoning module built on trachea segmentation to define an RIJL-containing subregion, and a postprocessing function to improve the segmentation. The details are given below.

### 3.1. Dataset

This retrospective study received approval from our institutional review board (IRB). The study cohort included patients from the medical and surgical ICUs within our hospital system between April 2018 and September 2019. Portable anteroposterior CXRs were acquired from adult patients with RIJL. All data were anonymized to meet IRB compliance requirements. CXR images were manually annotated by trained imaging analysts using an in-house developed imaging research platform [31], followed by verification from radiologists. The course of the RIJL was traced. A total of 937 CXR images were annotated and divided into 750 for training and 187 for testing (approximately 80%:20%). All CXR images were de-identified.

### 3.2. Deep Learning Segmentation System

**Figure 2:**
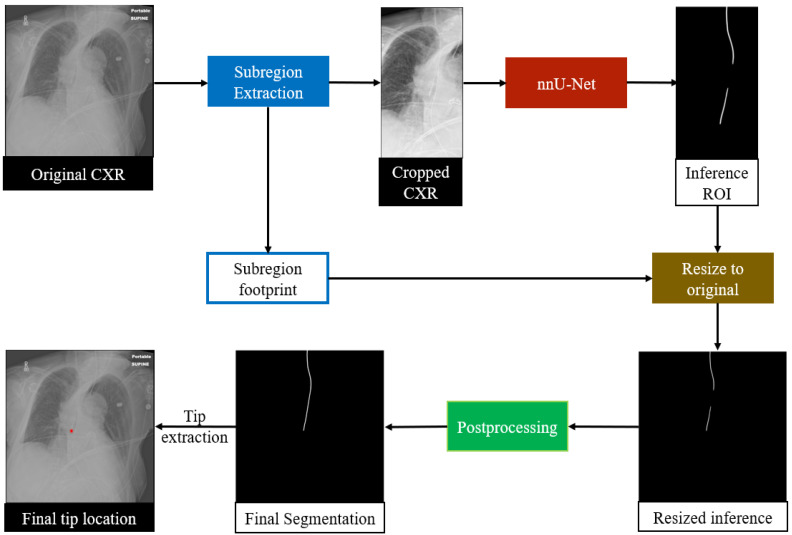
The overall framework for segmenting RIJL. The original CXR images are cropped based on trachea segmentation and then input to nnU-Net for training and inference, respectively. The prediction from nnU-Net is then resized to the original scale, followed by the Bidirectional Connect postprocessing step to enhance segmentation. The white lines indicate the segmentation masks. Finally, the RIJL segmentation mask is used to extract the tip location, as highlighted by the red dot.

An overview of the proposed RIJL segmentation system is displayed in Figure 2. The deep learning (DL) system was built on SimpleMind [32], an open-source Cognitive AI software platform for medical image analysis. SimpleMind enables users to create computer vision applications by embedding deep neural networks within a knowledge base. The knowledge base describes objects to be identified and relationships between them in the image. Additionally, spatial reasoning agents are provided to define search areas and check CNN outputs, e.g., defining a crop region for the RIJL relative to the trachea landmark. SimpleMind dynamically chains multiple software agents together to perform image preprocessing, CNN inferencing, and postprocessing. The software is available on its GitLab repository (https://gitlab.com/sm-ai-team/simplemind (accessed on 1 December 2024).

In this study, the trachea and the RIJL are the two target structures for segmentation. The trachea is segmented first, and its segmentation output is used for spatial reasoning to define a relevant region that guides the segmentation of the RIJL. As a result, a cropped dataset was generated to train and test the segmentation network. Further details of trachea guidance are provided in Section 3.3.

We selected nnU-Net to configure the segmentation network. In contrast to many contemporary deep learning-based segmentation frameworks that require expert-driven, labor-intensive manual parameter tuning for new tasks, nnU-Net offers a systematic, automated configuration approach that covers the entire segmentation pipeline, including preprocessing, neural network topology, training, and postprocessing, to adapt to new datasets. Therefore, we chose nnU-Net as the backbone of our system to segment the RIJLs in CXRs. We used the processed images generated from the trachea segmentation guidance for network training and inference. In the process of cropping images, a subregion footprint (i.e., the 2D coordinates of the upper-right vertex and the dimensions of the bounding box) was saved to resize the prediction back to the original scale later. The 2D U-Net configuration of nnU-Net was used with a GPU memory constraint of 11 GB for each fold in cross-validation.

In nnU-Net, the segmentation pipeline is configured based on three steps: (1) fixed parameters, such as architecture template, training schedule, and loss function, are unchanged; (2) rule-based parameters, such as network topology and batch size, are adapted to dataset properties and GPU memory constraint; (3) empirical parameters, including best configuration selection and postprocessing, are selected via cross-validation. A more detailed description of the design principles can be found in the Methods section in [6].

To preprocess the images, nnU-Net created a dataset fingerprint that captures image properties, including modality, distribution of spacings, and number of classes. In addition, the mean and standard deviation (SD), as well as the 0.5 and 99.5 percentiles of the foreground pixel intensities, were computed over all training samples. According to the dataset fingerprint, a subsequent pipeline fingerprint, which is essentially a combination of the fixed and rule-based parameters mentioned above, was set to cover all relevant parameters to configure the network architecture as well as the training process. During training and inference, each CXR image is normalized by z-scoring, i.e., a subtraction by its mean intensity value followed by a division by its SD. The target spacing was the median spacing of all training samples. To ensure large patch sizes that would capture more contextual information for network training, the batch size was set to 2. The dataset and pipeline fingerprints are summarized in Table 1.

A 2D U-Net-like architecture was configured, depending upon patch size and target spacing. The network was trained for 1000 epochs, with a loss function that is a sum of cross-entropy and Dice loss. Various data augmentation techniques were applied during training, including rotations and scaling, Gaussian noise and blur, brightness and contrast adjustment, low resolution simulation, Gamma correction and mirroring. Five-fold cross validation was applied on 750 samples for training, and 187 samples were held out for testing. When training was finished, the five models from the five folds were ensembled to inference on test samples with a sliding window approach, in which the window size equals the training patch size.

The entire segmentation framework was applied on the cropped dataset. To obtain a baseline performance, nnU-Net was also trained and tested on the original dataset. For some samples, the segmentation inference did not have a contiguous ROI, but multiple line fragments, and hence further postprocessing was required. There were two postprocessing methods applied, selecting the largest connected component and Bidirectional Connect—the former is simply a selection based on the size of ROIs, and the latter is explained in Section 3.4.

### 3.3. Trachea-Guided Spatial Reasoning

**Figure 3:**
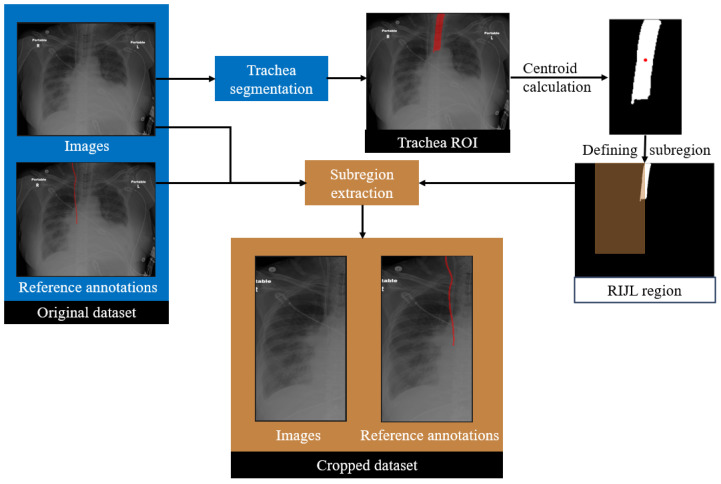
The workflow of subregion extraction guided by trachea segmentation. In Trachea ROI, the red region indicates trachea segmentation, the mask of which is marked with white in the two subsequent images. The red line indicates the reference annotations in Cropped dataset.

In the proposed framework, a trachea segmentation model, trained and tested on CXR images, was utilized to guide the RIJL segmentation, as shown in Figure 3. The trachea in each CXR image was segmented first using this model. From the segmentation output, the largest connected component was selected as the final region of interest (ROI). The 2D coordinates of the trachea ROI centroid were then calculated. Based on our knowledge of the typical location and the size of RIJL in a CXR image, a bounding box with dimensions of 300 mm in height and 150 mm in width, positioned to the right of the trachea, was defined. The bounding box was multiplied with the original image and ground truth annotation to extract a cropped region. This cropped region constituted a new dataset, referred to as the cropped dataset, whereas the original images are referred to as the original. Each cropped image was visually inspected to confirm coverage of a relevant region where an RIJL was likely to be present.

### 3.4. Postprocessing

Segmenting an RIJL in a CXR presents several challenges. First, the foreground pixels are significantly outnumbered by background pixels, making pixel-wise predictions highly prone to false positives. Additionally, similar line-like structures in the images further increase the risk of misclassification. Most critically, when an RIJL overlaps with or lies adjacent to another catheter, distinguishing between them becomes difficult during both CNN inference and postprocessing. Consequently, developing a customized postprocessing algorithm is crucial to enhance segmentation accuracy.

**Figure 4:**
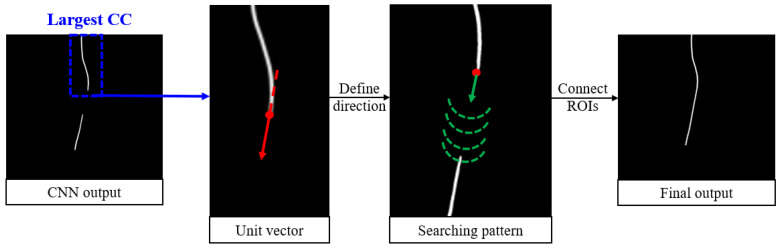
A graphical illustration of the Bidirectional Connect algorithm. For simplicity, a test sample is presented where only the inferior point of the largest connected component is used as the starting point. The dashed semicircles in the searching pattern are enlarged for clarity.

To address these challenges, a postprocessing algorithm called Bidirectional Connect was developed and is displayed in Figure 4. It begins by identifying the largest connected component (CC) among all regions of interest (ROIs) in the CNN output. These endpoints are determined by skeletonizing the largest CC and selecting the superior and inferior points. From there, the algorithm searches for nearby ROIs starting from the endpoint(s) of the largest CC. The search path is guided by a unit vector calculated based on the endpoint and its nearest neighbor. At each step along the search path, a semicircle is scanned around the current point to locate the next point along the trajectory. Any ROIs encountered along this path, in addition to the largest CC, are added to a new array. If a gap exists between two ROIs, the intermediate points are filled using linear interpolation. Finally, the connected curve is dilated with a disk-shaped kernel to ensure consistent line width. The output of this postprocessing module is a smooth, continuous curve that accurately connects the relevant line-like ROIs.

### 3.5. Evaluation Metrics

The performance of the proposed RIJL segmentation and tip localization system was evaluated quantitatively with two different metrics. Since RIJL is a thin tubular object that can be more accurately characterized as a boundary rather than a two-dimensional region, a boundary distance-based metric provides a better measure of the alignment between the RIJL segmentation output and the ground truth compared to overlap-based metrics, such as the Dice Similarity Coefficient (DSC), that lack robustness to small variations. Therefore, to evaluate the RIJL course segmentation performance, we used the average symmetric surface distance (ASSD) [33], which is defined as the average of the distances from the boundary of the segmentation output to the boundary of the ground truth. The formula to calculate ASSD is represented in Equation (1) below:

(1) $$ASSD\left(B_{1},\;B_{2}\right)=\frac{1}{\left|B_{1}\right|+\left|B_{2}\right|}\left(\sum_{x\in B_{1}}d(x,\;B_{2})+\sum_{y\in B_{2}}d(y,\;B_{1})\right)$$

where $B_{1}$ and $B_{2}$ are the boundaries of the segmentation output and the ground truth; *d*(*x*, *B*_2_) represents the shortest distance from a point *x* in $B_{1}$ to boundary $B_{2}$; $\left|B_{1}\right|$ and $\left|B_{1}\right|$ are the number of points on boundaries $B_{1}$ and $B_{2}$, respectively.

To evaluate the tip detection accuracy, the tip positions were extracted from the RIJL course segmentation and the ground truth. Specifically, the coordinates of the most inferior point within the RIJL region of interest were calculated and defined as the tip. The tip distance was determined as the Euclidean Distance between the two coordinates, adjusted by image spacings in the vertical and horizontal axes, respectively.

## 4. Results

The RIJL segmentation system, as described above, comprises a trachea-guided subregion extraction module, a segmentation network configured with nnU-Net, and a postprocessing function called Bidirectional (BD) Connect. This system was implemented, trained on 750 cropped CXR images, and tested on 187 cropped images. A resizing function was then applied to restore the full-scale segmentation output. For comparative analysis, additional experiments were conducted using different combinations of image scales and postprocessing methods.

### 4.1. Quantitative Analysis

Table 2 presents a summary of the quantitative results from the experiments on the test set. The segmentation network was trained and tested using both original and cropped CXR datasets. As a baseline, the original images were used to train and test the network, which generates the deep learning (DL) on original results. The segmentation results from the cropped images were projected back to the original scale, producing the DL on cropped results. Postprocessing was applied to further improve the segmentation performance: the largest connected component (CC) and BD Connect were applied to the *DL on* cropped results, adding two more rows to Table 2.

The mean and standard deviation (SD) of the mean ASSD and the tip distances, between the final segmentation and the ground truth, were computed on 187 test samples. The proposed segmentation method of this work, DL on cropped + BD Connect, outperforms all other methods in both RIJL course segmentation and tip detection. A statistically significant difference was observed (*p* < 0.05) when comparing the proposed method to the baseline nnU-Net results from the original dataset. The difference between the mean tip distances of DL on cropped + BD Connect and the baseline method was not statistically significant, but the lower mean and SD values indicate an improvement on the accuracy and precision of tip localization.

### 4.2. Visualization Results

**Figure 5:**
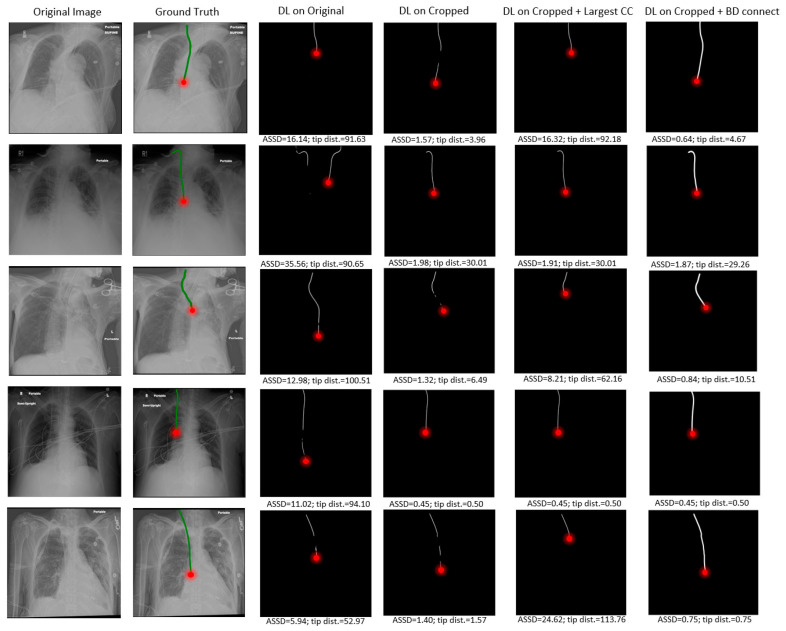
Examples from the test results, with each row corresponding to a single sample. In the Ground Truth images, the RIJL is marked in green. In each image, the red dot indicates the tip of the RIJL. The Bidirectional (BD) Connect postprocessing function applies a dilation step after connecting relevant ROIs—this step compensates for skeletonization, resulting in segmentation ROIs that appear thicker and brighter. The corresponding ASSD and tip distance in [mm] are given below each inference result.

Several visual examples of the test results from the different RIJL segmentation methods are collected and displayed in Figure 5. In the DL on original column, it is evident that the network’s prediction of RIJL is interfered by other tubular objects in the image, leading to over- and under-segmentation. Since the tip localization is dependent on the RIJL segmentation mask, the inaccurate segmentation masks cause large errors in tip detection. On the contrary, in the DL on cropped column, the network can focus on the RIJL region and capture a more complete shape of it as the final segmentation. Moreover, even when the cropped segmentation is fragmented (i.e., having multiple lines as seen in rows 1, 3, and 5), the tip can still be accurately located.

Comparing the columns of DL on cropped and DL on cropped + BD Connect, the capability of the BD Connect function is shown. BD Connect was able to connect the largest CC of the segmentation output to relevant ROIs by adapting the direction of the searching pattern to the shape of the largest CC, thus enhancing the RIJL segmentation performance.

**Figure 6:**
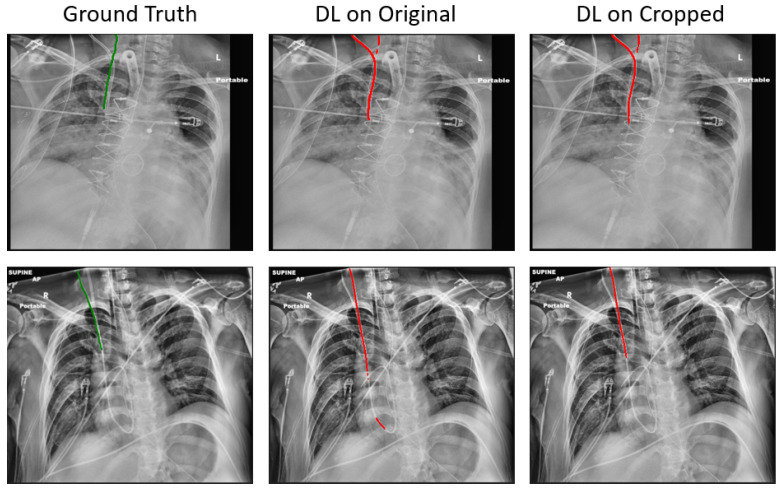
Representative test samples where the RIJL segmentation failed, with each row representing a single sample. The images were processed with CLAHE and overlaid with Ground Truth, DL on original and cropped segmentation outputs, respectively. The ground truths and segmentation outputs are marked with green and red, respectively.

Compared to the baseline, the proposed method has reduced the mean ASSD from 2.72 mm to 1.41 mm, and the mean tip error from 11.27 mm to 8.29 mm, on 187 held-out test samples. Notwithstanding these promising results, in certain samples, correctly segmenting the RIJL remains a challenge. As illustrated in Figure 6, other line-like, tubular objects, with strong visibility in the CXR images, can still mislead the final prediction. In the two instances given, both the original and cropped DL methods failed to segment the RIJL correctly, instead identifying a different structure. Moreover, the problem arises when another line is nearly overlapping with the RIJL, as seen in Figure 6 images, making it extremely difficult for a neural network to distinguish between them.

Given that this is the first study specifically designed to focus on the accurate segmentation of RIJL in CXRs, identifying prior studies for a direct and fair comparison is challenging. However, a few previously mentioned works may serve as a basis for comparative analysis. In [19], 450/150 images were used to train/test their PICC tip detection models and the final best performing model achieved an absolute tip distance of 3.10 ± 2.03 mm. Yu et al. [24] trained and tested their PICC segmentation models with 300 and 48 images, respectively, and their model achieved a DSC of 0.58 ± 0.01. Boccardi et al. [14] attempted instance segmentation in CXRs with a mixture of CVCs and Swan-Ganz Catheters (SWGs); with 8877 CXRs and 80%/20% for training/testing, they achieved a DSC of 0.739 ± 0.009. In our study, the DSC was calculated on the segmentation results of 187 test samples, yielding a mean ± standard deviation of 0.724 ± 0.150.

## 5. Discussion

This study proposed a novel anatomical landmark-guided deep learning framework to segment the RIJLs, the most applied type of CVCs, in CXR images. Since an RIJL is typically placed to the right of the trachea, the trachea serves as a valuable reference landmark for RIJL localization. In this work, a well-trained trachea segmentation network was used to identify the trachea region in each image of our CXR dataset. Subsequently, the segmentation output was employed to extract a local region that is most likely to contain an RIJL. The extracted regions constituted a new dataset, referred to as the cropped set, which was used to train and test an nnU-Net-based segmentation network. The segmentation output was then rescaled to the original image dimensions. Following a customized postprocessing step called Bidirectional Connect, the final segmentation was produced. This methodology demonstrated its effectiveness by achieving improved segmentation and tip localization performances, measured by the mean average symmetric surface distance (ASSD) and mean tip distance, as shown in Table 2. The promising results indicate that, with the incorporation of an existing accurate anatomical landmark segmentation, it is possible to improve the segmentation performance of related objects.

CVC tip positioning is clinically important as it indicates the efficacy of catheterization and patient safety. Consequently, several studies were conducted to classify the status of tip placement [4,9]. To enhance reliability and detection accuracy, direct localization of the CVC tip has gained popularity. However, due to the tiny footprint of the CVC tip in CXR images, treating it as an object detection problem presents substantial challenges. Instead, researchers have opted to segment the catheter first and then use this segmentation to help locate the tip [19,20,21]. Similarly, this work contributes to the RIJL tip detection by first segmenting the line and extracting the tip. The methodology and results represent a significant step toward clinical translation.

In this work, two notable aspects of nnU-Net are worth mentioning. First, nnU-Net’s built-in postprocessing method is the non-largest component suppression, which progressively removes smaller connected components to increase the Dice Coefficient during cross-validation. This approach performs adequately for the most part but is prone to errors when more complex decisions need to be made. Secondly, after cropping, the image spacing was changed from 0.15 to 1.0 mm. This adjustment may influence the patch size that nnU-Net configures: for cropped images, the patch size was 2048 × 768, while for original images, it was 1024 × 1536. A deeper investigation into the relationship between image spacing and patch size in the nnU-Net framework is warranted.

The segmentation performance was evaluated with the average symmetric surface distance (ASSD), as opposed to the Dice Similarity Coefficient (DSC), used in other CVC segmentation studies [5,14,22]. DSC, though a popular metric to evaluate segmentation performance in the field of deep learning, is not well suited for RIJL segmentation for several reasons. First, the thin line structures of RIJL occupy a small portion of the overall image, making DSC highly sensitive to minor inaccuracies that lead to large drops in DSC. Additionally, background pixels disproportionately influence DSC, further reducing its reliability. Second, RIJL requires precise localization, especially when overlapping with or adjacent to other line-like structures. While DSC might indicate a reasonable value when the segmentation is in the vicinity of the ground truth, ASSD offers higher precision, making it a more dependable metric in this context. Finally, given the small area of RIJL, DSC tends to underestimate false negatives, which may fail to effectively account for discontinuity in the line structure. In summary, ASSD provides a more reliable evaluation of RIJL segmentation compared to DSC.

Although not a direct comparison, our proposed segmentation system demonstrates competitive performance relative to two state-of-the-art catheter segmentation studies [14,24] in CXRs. The tip detection performance is suboptimal compared to the PICC tip detection in [20], highlighting the need for future work to enhance RIJL tip detection.

This study has a few limitations. First, it only evaluated the performance of nnU-Net configured networks across different image dimensions. A more comprehensive comparison with other published CVCs or catheter segmentation methods is needed. Second, the study was conducted on a relatively small in-house dataset. Future work should investigate the effectiveness and robustness of the method on larger datasets, such as CLiP [28], to better assess its generalizability and performance across diverse variations.

## 6. Conclusions

The goal of this work was to leverage anatomical landmark segmentation to guide RIJL segmentation and tip localization. Specifically, a deep learning system was developed within the SimpleMind Cognitive AI framework, incorporating an anatomical landmark segmentation for guidance, an nnU-Net-based RIJL segmentation network, and customized postprocessing to enhance segmentation performance. The proposed method demonstrated its effectiveness through improved performances in RIJL course segmentation and tip detection, evaluated with the average symmetric surface distance (ASSD) and absolute tip distance, respectively.

## Figures and Tables

**Table 1:** Dataset and pipeline fingerprints from nnU-Net configurations.

Parameters	2D U-Net on Cropped CXR	2D U-Net on Full CXR
Target spacing (mm)	1.0 × 1.0	0.15 × 0.15
Median image size	2303 × 1000	2336 × 2836
Intensity normalization: mean, SD	Z-score: µ = 125.8, σ = 34.9	Z-score: µ = 125.9, σ = 35.0
Patch size	2048 × 768	1024 × 1536
Batch size	2	2
GPU memory limit (GB)	11	11

**Table 2:** Quantitative comparison of different RIJL segmentation methods on the test set (*N* = 187), with numerical values as mean ± SD and 95% confidence intervals, respectively.

Segmentation Methods	ASSD [mm]		Tip Distance [mm]	
	Mean ± SD	95 Conf. Interval	Mean ± SD	95 Conf. Interval
DL on Original	2.72 ± 6.36	(1.81, 3.63)	11.27 ± 25.33	(7.64, 14.90)
DL on Cropped	1.43 ± 4.12	(0.84, 2.02)	8.91 ± 18.72	(6.22, 11.59)
DL on Cropped + largest CC	1.78 ± 4.62	(1.12, 2.44)	10.71 ± 21.0	(7.16, 13.18)
DL on Cropped + BD connect	1.41 ± 3.83	(0.87, 1.96)	8.29 ± 16.76	(5.89, 10.69)

## Data Availability

The datasets presented in this article are not readily available due to University policies. Requests to access the datasets should be directed to Siyuan.
